# Significance of Photosynthetic Characters in the Evolution of Asian *Gnetum* (Gnetales)

**DOI:** 10.3389/fpls.2019.00039

**Published:** 2019-02-05

**Authors:** Nan Deng, Chen Hou, Caixia Liu, Minghe Li, Igor Bartish, Yuxin Tian, Wei Chen, Changjian Du, Zeping Jiang, Shengqing Shi

**Affiliations:** ^1^State Key Laboratory of Tree Genetics and Breeding, Key Laboratory of Tree Breeding and Cultivation of State Forestry Administration, Research Institute of Forestry, Chinese Academy of Forestry, Beijing, China; ^2^Hunan Academy of Forestry, Changsha, China; ^3^Hunan Cili Forest Ecosystem State Research Station, Cili, China; ^4^School of Life Sciences, Sun Yat-sen University, Guangzhou, China; ^5^Department of Ecology, Environment and Plant Sciences, Stockholm University, Stockholm, Sweden; ^6^College of Landscape Architecture, Fujian Agriculture and Forestry University, Fuzhou, China; ^7^Department of Genetic Ecology, Institute of Botany, Academy of Sciences of Czech Republic, Praha, Czechia; ^8^Institute of Forest and Ecology Protection, Chinese Academy of Forestry, Beijing, China

**Keywords:** photosynthesis, chloroplast genomes, phylogeny, seed plants evolution, gymnosperms

## Abstract

*Gnetum* is a genus in the Gnetales that has a unique but ambiguous placement within seed plant phylogeny. Previous studies have shown that *Gnetum* has lower values of photosynthetic characters than those of other seed plants, but few *Gnetum* species have been studied, and those that have been studied are restricted to narrow taxonomic and geographic ranges. In addition, the mechanism underlying the lower values of photosynthetic characters in *Gnetum* remains poorly understood. Here, we investigated the photosynthetic characters of a Chinese lianoid species, i.e., *Gnetum parvifolium*, and co-occurring woody angiosperms growing in the wild, as well as seedlings of five Chinese *Gnetum* species cultivated in a greenhouse. The five *Gnetum* species had considerably lower values for photosynthesis parameters (net photosynthetic rate, transpiration rate, intercellular CO_2_ concentration, and stomatal conductance) than those of other seed plant representatives. Interrelated analyses revealed that the low photosynthetic capacity may be an intrinsic property of *Gnetum*, and may be associated with its evolutionary history. Comparison of the chloroplast genomes (cpDNAs) of *Gnetum* with those of other seed plant representatives revealed that 17 coding genes are absent from the cpDNAs of all species of *Gnetum*. This lack of multiple functional genes from the cpDNAs probably leads to the low photosynthetic rates of *Gnetum*. Our results provide a new perspective on the evolutionary history of the Gnetales, and on the ecophysiological and genomic attributes of tropical biomes in general. These results could also be useful for the breeding and cultivation of *Gnetum*.

## Introduction

The Gnetales comprises three genera i.e., *Ephedra* L., *Welwitschia* Hook. f., and *Gnetum* L, and forms a monophyletic group, as indicated by morphological and molecular data (Price, 1996; Doyle, 1998; Rydin and Korall, 2009). The Gnetales are morphologically different from other gymnosperms and their phylogenetic placement within seed plants is unclear (Doyle and Donoghue, 1986; Friedman, 1998; Burleigh and Mathews, 2004). Previous paleobiological, palynological, morphogenetic, and anatomical studies have suggested that there is a close relationship between the Gnetales and angiosperms, i.e., the “anthophyte” hypothesis (Crane, 1985; Doyle and Donoghue, 1986, 1992). However, molecular phylogenies generated in last decade have placed the Gnetales as a sister group to the Pinaceae (the “gnepine” hypothesis), conifers (the “gnefers” hypothesis, Soltis et al., 2002; Ickert Bond and Wojciechowski, 2004; Wu et al., 2007; Zhong et al., 2010; Gong et al., 2016; Ickert Bond and Renner, 2016; Mao et al., 2017; Ran et al., 2018; Wan et al., 2018), or other seed plants (Chen et al., 2016). The ambiguous placement of the Gnetales within seed plant phylogenies is ascribed to the lack of homologous features among different plant groups.

*Gnetum* comprises around 40 species. Most *Gnetum* species are woody climbers and a few species are shrubs and trees (Markgraf, 1930, 1951, 1965; Hou et al., 2015). The genus has a broad distribution in lowland mixed and dense areas of pantropical forests. Leaves of *Gnetum* are rich in bioactive compounds, e.g., flavonoids and stilbenes, which have remarkable medical effects (Deng et al., 2016, 2017). Phylogenetic studies based on molecular data have shown that South American *Gnetum*, African *Gnetum*, and Asian *Gnetum* constitute the three major clades of the genus (Won and Renner, 2006; Hou et al., 2016). Within Asian *Gnetum*, two arborescent species *Gnetum gnemon* L. and *G. costatum* K. Schum comprise a sister clade to other (lianoid) Chinese and Indo-Malayan species (Hou et al., 2015, 2016). The phylogenetic relationships and delimitations of Chinese lianoid *Gnetum* have been resolved in a recent study based on morphological and molecular data (Hou et al., 2016).

*Gnetum* species are characterized by such traits as decussate leaves, pinnate leaf veins, and the presence of vessels in stems, all of which resemble characters of angiosperms (Markgraf, 1930). However, two previous studies found that the photosynthetic and transpiration capacities of *Gnetum* are considerably lower than those of other seed plants. For example, the photosynthetic capacities of four *Gnetum* species, i.e., *G. costatum, G. gnemon*, and *Gnetum latifolium* Blume, and one unidentified lianoid species were found to be consistently lower than those of co-occurring angiosperms in tropical rainforest in Papua New Guinea (Feild and Balun, 2008). The results showed that all the studied species of *Gnetum* had lower photosynthetic rates in terms of stomatal conductance and transportation of stem water (Feild and Balun, 2008). The other case was the low values of photosynthetic characters detected in seedlings of *Gnetum leyboldii* Tul. under greenhouse conditions (Celis and Avalos, 2013). The results of those two studies suggested that the presence of xylem vessels, broad net-veined leaves, and lianoid habitats do not necessarily indicate highly opportunistic and light-demanding ecophysiological capacities in *Gnetum* (Feild and Balun, 2008; Celis and Avalos, 2013).

Nevertheless, it would be premature to draw a robust conclusion that low photosynthetic capacity is a typical feature of *Gnetum*, since relatively few species have been studied in detail. To the best of our knowledge, the photosynthetic characters of Chinese lianoid *Gnetum* species have not been analyzed yet. Besides, the intrinsic mechanisms underlying low values of photosynthetic characters of *Gnetum* are poorly understood. In the present study, the first aim was to compare several photosynthetic characters between Chinese lianoid *Gnetum* and their co-occurring angiosperms, conifers, and *Ginkgo* in the wild, as well as under different experimental conditions. The second aim was to compare changes in photosynthetic characters through time among Chinese lianoid species. The last aim was to use photosynthetic character data and sequences of cpDNAs to determine patterns of photosynthetic evolution in the context of seed plant phylogeny. The overall aim of this study was to better understand the role of photosynthetic function in the evolution of the Gnetales using integrated experimental plant physiological, genomic, phylogenetic, systematic, and ecological analyses. The obtained knowledge could be beneficial for the breeding and cultivation of *Gnetum*.

## Materials and Methods

### Study Sites and Samples

Field experiments were conducted at Fuzhou Forest Park (E 119.29°, N 26.15°) in Fujian province, China in the middle of September 2016. The habitats of Chinese lianoid *Gnetum* are in the lowland area in mixed and dense subtropical forests. An open site with natural light was selected in a forest-edge zone in the park, where *Gnetum parvifolium* (Warb.) W. C. Cheng is distributed and grows well alongside several other plants representing some of the main lineages of land plants. Photosynthetic parameters in three individuals of *G. parvifolium* were measured using the LI-6400 portable photosynthesis system (LI-COR Inc. Lincoln, NE, United States). Photosynthetic parameters were also measured for three selected co-occurring plants; *Lonicera japonica* Thunb., *Styrax confusus* Hemsl., and *Pteris vittata* L. (Table 1). These plants were selected on the basis of the following criteria: (1) they were representatives of main lineages of land plants, i.e., angiosperms and ferns (but not gymnosperms, which grew at heights out of reach at the site); (2) they were representatives of liana with broad leaves like those of *Gnetum*; (3) cpDNA sequences were available for them or for other species in their genus.

**Table 1:** Taxonomy, ecology, ages, and sample sizes of *Gnetum* and other seed plants used for measurements of photosynthetic parameters.

In wild								
**Species**	**Family**	**Order**	**Location**	**Environment**	**Number**	**Status**	**Age**	**Main co-occurring species**
*Gnetum parvifolium* (Warb.) W.C.Cheng	Gnetaceae	Gnetales	Open and high-light zone Forest edges near roadsides	Temperature: 30°C; photosynthetic photon flux density (PPFD) 850-950 μmol⋅m^-2^⋅s^-1^ and the carbon dioxide level around 400 μmol⋅mol^-1^	3	Liana	>10 years	*Pinus massoniana* Lamb., *Cinnamomum camphora* (L.) J. Presl, *Loropetalum chinensis* (R. Br.) Oliv.
*Lonicera japonica* Thunb	Caprifoliaceae	Dipsacales			3			*Pinus massoniana* Lamb., *Loropetalum chinensis* (R. Br.) Oliv.
*Styrax confusus* Hemsl	Styracaceae	Ericales			8	Shrub		*Loropetalum chinensis* (R. Br.) Oliv.
*Pteris vittata* L.	Pteridaceae	Polypodiales			>20	Herbal	<1 year	*Loropetalum chinensis* (R. Br.) Oliv.
**In greenhouse**								
*Ginkgo biloba* L.	Ginkgoaceae	Ginkgoales	In greenhouse open and high-light zone	Temperature: 27–30°C; photosynthetic photon flux density (PPFD) around 800 μmol m^-2^ s^-1^ and the carbon dioxide level 350–400 μmol mol^-1^	>10	Shrub	2 years	
*Gnetum gnemon* L.	Gnetaceae	Gnetales				Tree and shrub	2 years	
*Gnetum luofuense* C.Y.Cheng						Liana	2 and 5 years	
*Gnetum montanum* Markgr.							2 and 5 years	
*Gnetum parvifolium* (Warb.) W.C.Cheng							5 years	
*Gnetum pendulum* C.Y.Cheng							2 years	
*Pinus tabuliformis* Carr.	Pinaceae	Pinales				Shrub	2 years	
*Populus tomentosa* Carrière								
*Salix babylonica* L.	Salicaceae	Malpighiales						
*Sasa argenteostriata* (Regel) E.G. Camus	Poaceae	Poales						

At the field site (8 × 2 m), three individuals of *G. parvifolium* and co-occurring species were randomly selected, but all were at least 2 m away from other individuals of the same species. The photosynthetic characters of selected plants were measured at 8:30–10:00 a.m., and measurements were repeated three times on 3 consecutive days, with the ambient temperature around 30°C, photosynthetic photon flux density (PPFD) between 850 and 950 μmol m^-2^ s^-1^ and carbon dioxide (CO_2_) level around 400 μmol mol^-1^. Because *Gnetum* are evergreen trees, we measured photosynthetic characters of old leaves (at the base) and young leaves (usually at the top) in the current-year branches of *G. parvifolium*. For the other three plant species, we measured photosynthetic parameters of fully opened leaves (functional leaves) at similar internodes from the shoot tip. The final values of photosynthetic parameters were the average of values measured over the 3 days of observations.

We also conducted experiments at 8:30–10:00 a.m. in the greenhouse of the Chinese Academy of Forestry, Beijing, China from June to September 2016. The conditions in the indoor environment were adjusted to levels similar to those at the field site: i.e., temperature around 27°C, PPFD around 800 μmol m^-2^ s^-1^; and CO_2_ around 350–400 μmol mol^-1^. We measured photosynthetic characters of 2-year-old seedlings of one arborescent species (i.e., *Gnetum gnemon*), and four lianoid species (i.e., *Gnetum pendulum* C. Y. Cheng, *Gnetum montanum* Markgr., *G. parvifolium*, and *Gnetum luofuense* C. Y. Cheng). We compared photosynthetic characters between 2-year-old and 5-year-old seedlings of *G. montanum* and *G. luofuense* to control for possible ontogenetic shifts in these characters. We also measured photosynthetic characters in four 2-year-old seedlings of *Ginkgo biloba* L., *Pinus tabuliformis* Carr., *Populus* × *tomentosa* Carrière, *Salix babylonica* L., and *Sasa argenteostriata* (Regel) E.G. Camus. The photosynthetic characters of *P. tabuliformis* were measured in a needle chamber. These plants were selected on the basis of the following criteria: (1) they were representatives of main lineages of seed plants, i.e., angiosperms and gymnosperms; and (2) cpDNA sequences were available for them or for a member of their genus. Table 1 summarizes details of the taxonomy, ecology, ages, and sample sizes of all plants used in this study. The photosynthetic characters of *Gnetum* and other seed plant representatives were measured from June to September, which was the fruiting season of *Gnetum*. During this time, the photosynthetic characters were most likely to reach their peaks and reflect the photosynthetic capacity. We measured the photosynthetic characters of four individuals of each species of *Gnetum* and obtained the mean value for each month. Identical measurements of photosynthetic characters were performed in four individuals each of *G. biloba, P. tabuliformis, P. tomentosa, S. babylonica*, and *S. argenteostriata*.

### Measurement of Photosynthetic Characters

Five photosynthetic characters were measured using the LI-6400 Portable Photosynthesis System: net photosynthetic rate (Pn) (reflecting the accumulation of photosynthetic products in plants); transpiration rate (Tr) (transport resistance of CO_2_ and water); intercellular CO_2_ concentration (Ci) (level of CO_2_ available for photosynthesis); stomatal conductance (Gs) (stomatal opening in proportion to transpiration); and leaf water deficit (Vpdl) (an index of transpiration). Water use efficiency (WUE) was obtained by dividing Pn by Tr. We measured relative chlorophyll content (Rc), indicative of percentage chloroplast content, using a SPAD-502 Plus portable chlorophyll meter (Konica Minolta, Osaka, Japan).

Light-/CO_2_-response curves reflect the responses of photosynthetic characters to light intensity (or CO_2_ concentration). These curves show the photosynthetic efficiency of plants across a photon flux gradient under different concentrations of light/CO_2_. In the greenhouse, we produced light/CO_2_ response curves for 2-year-old seedlings of the five *Gnetum* species i.e., *G. gnemon, G. luofuense, G. montanum, G. pendulum*, and *G. parvifolium* using the LI-6400 Portable Photosynthesis System (Li-Cor). The light-response curves were generated with a CO_2_ concentration of 400 μmol mol^-1^ in the greenhouse. For the light response curves, we adjusted the light intensity to a range of levels: 2000, 1800, 1500, 1200, 1000, 800, 600, 400, 200, 150, 100, 50, and 0 μmol m^-2^ s^-1^, as applied in an earlier study (Palliotti and Cartechini, 2015). Polynomial quadratic equations were calculated (Farquhar et al., 2001) with the best fit to the light-response data for light compensation point (LCP), light saturation point (LSP), maximum photosynthesis rate of light-response (AmaxL), dark respiration rate of light-response (RdL), and apparent quantum yield (AQY). The LCP is the light intensity when the synthesis and consumption of organic materials are equal. The LSP is the minimum light intensity when Pn values reach the maximum. The RdL is the Pn value when the light intensity is 0, and indicates the consumption of organic materials without photosynthesis. The AQY is the initial slope when light intensity ranges from 0 to 200 μmol m^-2^ s^-1^, and reflects the utilization efficiency of plants under weak light (Farquhar et al., 2001).

For the CO_2_-response curves, we set the light intensity at 1000 μmol m^-2^ s^-1^ in the greenhouse. We established a gradient of CO_2_ concentrations: 400, 200, 100, 0, 50, 100, 150, 200, 300, 400, 600, 800, 1200, 1500, and 2000 μmol mol^-1^, as described by Yin et al. (2016). We calculated polynomial quadratic equations with the best fit to the light-response data for CO_2_ compensation point (CCP), CO_2_ saturation point (CSP), maximum photosynthesis rate of CO_2_-response (AmaxC), carboxylation efficiency (CE), and dark respiration rate of CO_2_-response (RdC). A cubic polynomial was used to fit the curves of the five species of *Gnetum* in both the light-response and CO_2_-response curves. The meanings of the photosynthetic characters in the CO_2_ response curves are identical to those of the light response curves described above.

### Analyses of Detected Photosynthetic Characters

We performed principal component analyses (PCA) and cluster analyses of the five photosynthetic characters among the five *Gnetum* species and the other seed plant representatives. These analyses allowed us to explore the relationships among these species on the basis of variations in their photosynthetic characters. Data for the five photosynthetic characters, i.e., Pn, Tr, Ci, Gs, and Vpdl, were obtained for all compared species (see above) and standardized prior to analyses. In the PCA analyses, the Kaiser-Guttman criterion was applied for eigenvalue selection (Guttman, 1954; Kaiser, 2016). Six clustering methods, i.e., complete linkage agglomerative clustering, single linkage agglomerative clustering, unweighted pair-group method using arithmetic averages (UPGMA), unweighted pair-group method using centroids (UPGMC), weighted pair-group method using centroids (WUPGMA), and Ward’s minimum variance clustering, were applied using the same photosynthetic character data as used in the PCA. The application of six cluster methods was an exploratory analysis rather than a statistical test, and allowed us to compare the results of cluster analyses using different algorithms. We calculated the cophenetic correlation coefficient and statistic support for each clustering method using the *vegan* package implemented in R platform version 3.4.4 (R core team, 2016^1^).

### Comparison of Chloroplast Genomes of Selected or Related Plants

The cpDNAs of the 15 plants used in our photosynthetic analyses or belonging to the same genus were downloaded from Genbank ^2^. The accession numbers are shown in Table 2. The plants in these analyses were members of the Gnetales, *Ginkgo, Pinus*, five species of angiosperms, and *Pteridium* was the outgroup. We used the data matrix of seed plants (Ruhfel et al., 2014) supplemented by the cpDNAs of Chinese lianoid *Gnetum* (Hou et al., 2016). For each cpDNA, 78 conservative coding genes (listed in Supplementary Table S1) were chosen and aligned using MAFFT version 7.017 (Katoh et al., 2017) and visualized using mVISTA software (Mayor et al., 2000). Maximum likelihood trees were constructed based on concatenation of coding genes using the substitution model GTR+GAMMA implemented in RAxML version 7.2.8 (Stamatakis, 2006). In these analyses, we did not aim to re-evaluate the phylogenetic relationships among the Gnetales and other groups of seed plants. Instead, we reconstructed the phylogeny of selected seed plants as the evolutionary context for analyses of photosynthetic characters in this clade.

**Table 2:** Taxonomy of land plant representatives and GenBank accession numbers of their chloroplast genomes.

Order	Family	Genus	Species	Accession number
Dipsacales	Lonicera	*Lonicera*	*L. japonica*	GQ997381-GQ997463
Ephedrales	Ephedraceae	*Ephedra*	*E. equisetina*	NC_011954
Ericales	Styracaceae	*Styrax*	*S. grandiflorus*	NC_030539.1
Ginkgoales	Ginkgoaceae	*Ginkgo*	*G. biloba*	DQ069337-DQ069702 EU016963-EU016982
Gnetales	Gnetaceae	*Gnetum*	*G. parvifolium*	NC_011942 KX385191
Gnetales	Gnetaceae	*Gnetum*	*G. gnemon*	KX385188
Gnetales	Gnetaceae	*Gnetum*	*G. luofuense*	KX234236
Gnetales	Gnetaceae	*Gnetum*	*G. pendulum*	KX385198
Gnetales	Gnetaceae	*Gnetum*	*G. montanum*	KX385196
Malpighiales	Salicaceae	*Populus*	*P. trichocarpa*	NC_009143
Malpighiales	Salicaceae	*Salix*	*S. babylonica*	NC_028350.1
Pinales	Pinaceae	*Pinus*	*P. koraiensis*	NC_004677
Poales	Poaceae	*Sasa*	*S. veitchii*	KU569975
Polypodiales	Dennstaedtiaceae	*Pteridium*	*P. aquilinum*	NC_014348
Gnetales	Welwitschiaceae	*Welwitschia*	*W. mirabilis*	NC_010654

## Results

### Photosynthetic Characters of *Gnetum* in the Wild

Our results revealed that the mean Pn in *G. parvifolium* was 1.3 ± 0.33 μmol m^-2^ s^-1^ CO_2_, significantly lower than that in *S. confusus* (4.86 ± 0.08 μmol m^-2^ s^-1^), the lowest value detected among the four co-occurring species in the wild (Table 3). Similarly, the lowest values of Gs, Ci, and Tr were detected in *G. parvifolium*. However, the Vpdl and Rc values were significantly higher in *G. parvifolium* than in three co-occurring species. These results confirmed that *Gnetum* has a low photosynthetic capacity. To explore this in more detail, we conducted subsequent investigations of photosynthetic characters among five Chinese lianoid species under the same conditions in a greenhouse.

**Table 3:** Photosynthetic characters of *G. parvifolium* and three species of land plant representatives measured in the wild. Data are means ± standard deviation. Different letters in same column indicate significant difference (*p* < 0.05).

Species	Pn (μmol m^-2^ s^-1^ CO_2_)	Gs (mmol m^-2^s^-1^)	Ci (μmol mol^-1^)	Tr (g m^-2^ h^-1^)	VpdL (kPa)	Rc (%)
*Gnetum parvifolium*	1.30 ± 0.33a	0.01 ± 0.00a	164.64 ± 33.86a	0.20 ± 0.39a	2.09 ± 0.06a	57.86 ± 6.83a
*Lonicera japonica*	8.83 ± 0.24c	0.08 ± 0.01b	223.01 ± 15.79c	1.39 ± 0.04c	1.63 ± 0.08b	49.96 ± 4.11c
*Pteris vittata*	5.80 ± 0.41b	0.07 ± 0.00b	360.26 ± 11.08d	1.05 ± 0.02b	1.52 ± 0.04b	34.81 ± 4.92b
*Styrax confusus*	4.86 ± 0.08b	0.11 ± 0.00b	319.34 ± 2.68b	1.87 ± 0.1b	1.69 ± 0.04b	33.17 ± 1.82b

*Abbreviations of photosynthetic characters are as follows: Pn, net photosynthetic rate; Gs, stomatal conductance; Ci, intercellular CO_2_ concentration; Tr, transpiration rate; VpdL, leaf water deficit; Rc, relative chlorophyll content.*

### Light-Response Curves of *Gnetum*

**FIGURE 1:**
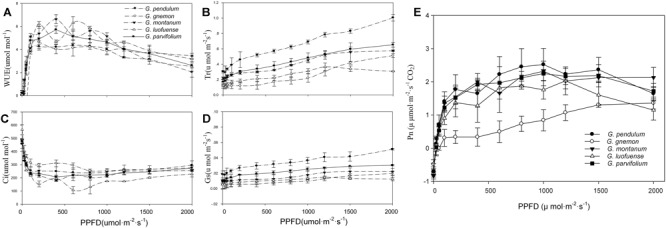
Light-response curves of five *Gnetum* species. Y-axes represent five photosynthetic characters (mean values ± standard deviation, *n* = 3), i.e., **(A)** photosynthetic rate (Pn), **(B)** stomatal conductance (Gs), **(C)** intercellular CO_2_ concentration (Ci), **(D)** transpiration rate (Tr), and **(E)** water use efficiency (WUE). X-axis represents photosynthetic photon flux density (PPFD).

As shown in the light-response curves, the Pn values plateaued at around 800 μmol m^-2^ s^-1^ among the four lianoid species, and slightly declined when the concentration of CO_2_ increased from 1.41 to 2.75 μmol m^-2^ s^-1^ (Figure 1A). In contrast, the Pn values of the arborescent species *G. gnemon* steadily increased when exposed to dense light (about 800 μmol m^-2^ s^-1^), but the values were considerably lower than most of those of other plants in the studied light intensity range (Figure 1A). The fitting degree (R^2^) ranged from 0.81 to 0.92 among the five species of *Gnetum*, indicating a good fit by cubic polynomial equations.

The values of Gs, Tr, and WUE were lower in *G. gnemon* than in the four lianoid species across most of the photosynthetic photon flux density range (Figure 1B–E). The four lianoid species and *G. gnemon* were similarly compared in terms of LCP, LSP, AmaxL, RdL, and AQY. The results indicated that the four lianoid species, in general, had greater photosynthetic potential than did *G. gnemon* (Table 4). Most of estimates for *G. gnemon* were significantly (*p* < 0.05) different from the estimates for other species (Table 4). *G. pendulum* had the highest photosynthetic capacity among the four lianoid species (Figure 1A–E).

**Table 4:** Photosynthetic characters of five *Gnetum* species in response to different light intensities. Data are means ± standard deviation. Different letters in the same column indicate significant difference (*p* < 0.05).

Species	LCP (μmol m^-2^ s^-1^)	LSP (μmol m^-2^ s ^1^)	AmaxL (μmol m^-2^ s^-1^CO_2_)	RdL (μmol m^-2^ s^-1^)	AQY
*Gnetum gnemon*	54.27 ± 12.88b	≥2000b	1.41 ± 0.12a	0.24 ± 0.01ab	0.004 ± 0.001a
*Gnetum luofuense*	28.50 ± 7.25a	1100.50 ± 200.34a	2.11 ± 0.31b	0.25 ± 0.02b	0.010 ± 0.002b
*Gnetum montanum*	16.36 ± 0.55a	950.25 ± 100.17a	2.31 ± 0.06b	0.17 ± 0.02a	0.010 ± 0.002b
*Gnetum parvifolium*	28.06 ± 12.43b	1099.67 ± 172.63a	2.28 ± 0.32b	0.28 ± 0.13a	0.010 ± 0.004a
*Gnetum pendulum*	16.39 ± 3.36a	950.25 ± 100.17a	2.75 ± 0.32c	0.16 ± 0.09a	0.010 ± 0.002b

*Abbreviations of photosynthetic characters are as follows: LCP, light compensation point; LSP, light saturation point; AmaxL, maximum photosynthesis rate of light-response; RdL, dark respiration rate of light-response; AQY, apparent quantum yield.*

### CO_2_-Response Curves of *Gnetum*

**FIGURE 2:**
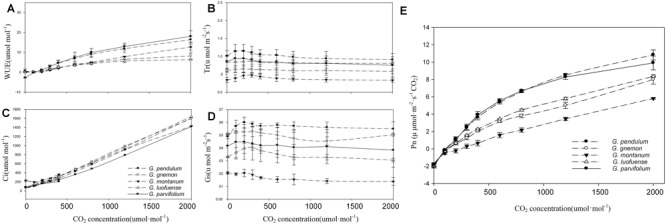
CO_2_-response curves of five *Gnetum* species. Y-axes represent five photosynthetic characters (mean values ± standard deviation, *n* = 3), i.e., **(A)** photosynthetic rate (Pn), **(B)** stomatal conductance (Gs), **(C)** intercellular CO_2_ concentration (Ci), **(D)** transpiration rate (Tr), and **(E)** water use efficiency (WUE). X-axis represents CO_2_ concentration.

As shown in the CO_2_-response curves, the Pn values among the five *Gnetum* species remarkably increased as the CO_2_ concentration increased, and reached a maximum value of 10.84 μmol m^-2^ s^-1^ (Figure 2A). *G. parvifolium* and *G. pendulum* had the highest Pn values in response to increased CO_2_, while *G. montanum* had the lowest (Table 5). The fitting degree (R^2^) ranged from 0.98 to 0.99 among the five species of *Gnetum*, indicating a very good fit by cubic polynomial equations.

**Table 5:** Photosynthetic characters of five *Gnetum* species in response to different concentrations of CO_2_. Data are means ± standard deviation. Different letters in the same column indicate significant difference (*p* < 0.05).

Species	CCP (μmol mol^-1^)	CSP (μmol mol^-1^)	AmaxC (μmol m^-2^ s^-1^CO_2_)	CE (μmol m^-2^ s^-1^)	RdC (μmol m^-2^ s^-1^)
*Gnetum gnemon*	167.55 ± 5.20a	≥2000	8.00 ± 0.02b	0.009 ± 0.0004b	1.582 ± 0.11ab
*Gnetum luofuense*	155.82 ± 17.05a	≥2000	8.36 ± 1.14b	0.010 ± 0.0004b	1.614 ± 0.23ab
*Gnetum montanum*	260.66 ± 54.70b	≥2000	5.82 ± 0.12a	0.006 ± 0.001a	1.401 ± 0.06a
*Gnetum parvifolium*	133.04 ± 11.31b	≥2000	10.71 ± 0.80a	0.014 ± 0.001a	1.810 ± 0.06a
*Gnetum pendulum*	129.12 ± 11.08a	≥2000	10.84 ± 0.83c	0.014 ± 0.001c	1.801 ± 0.05b

*Abbreviations of photosynthetic characters are as follows: CCP–CO_2_ compensation point; CSP–CO_2_ saturation point; AmaxC–maximum photosynthesis rate of CO_2_-response; CE–carboxylation efficiency; RdC–dark respiration rate of CO_2_-response.*

The five *Gnetum* species showed similar trends in Tr and Gs in response to increasing CO_2_ concentrations (Figure 2). *G. pendulum* had the highest Tr and Gs values across the studied range of CO_2_ concentrations, while *G. montanum* had the lowest. The results revealed that an increased concentration of CO_2_ in air could increase the Ci and WUE, but did not significantly affect Tr and Gs in *Gnetum* (Figure 2B,E).

### Monthly Variations in Photosynthetic Characters in *Gnetum* and Other Seed Plants

**FIGURE 3:**
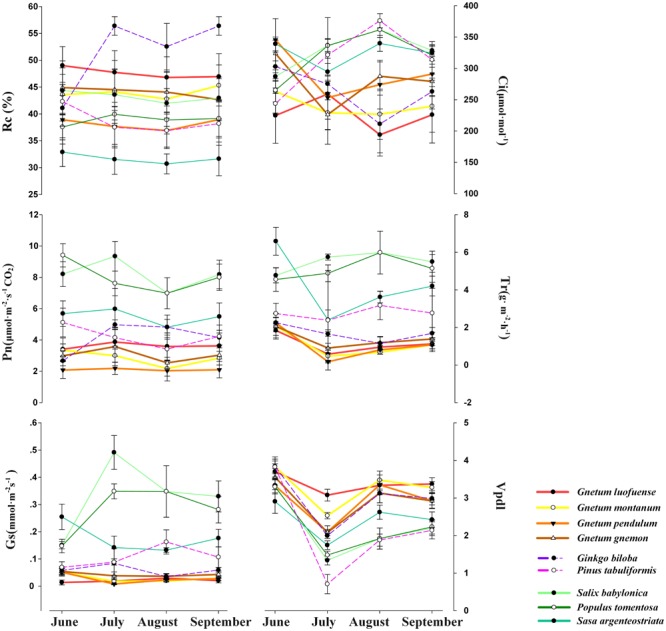
Comparisons of photosynthetic characters measured from June to September among four *Gnetum* species and five seed plant representatives. X-axes represent six photosynthetic characters (mean values ± standard deviation, *n* = 3), i.e., relative chlorophyll (Rc), intercellular CO_2_ concentration (Ci), photosynthetic rate (Pn), transpiration rate (Tr), stomatal conductance (Gs), and leaf water deficit (Vpdl).

The Tr, Pn, Gs, Vpdl, and Rc remained unchanged or only slightly changed from June to September among the five *Gnetum* species. However, the Ci values showed dramatic variations over this period of time (Figure 3). The values of photosynthetic characters such as Pn, Tr, and Gs were much lower in the five species of *Gnetum* than in the other seed plant representatives, i.e., *P. tomentosa, P. tabuliformis, S. babylonica*, and *S. argenteostriata*. However, the differences were minor among these seed plant groups in terms of Ci, Vpdl, and Rc (Figure 3).

### Principal Component and Cluster Analyses of Photosynthetic Characters

**FIGURE 4:**
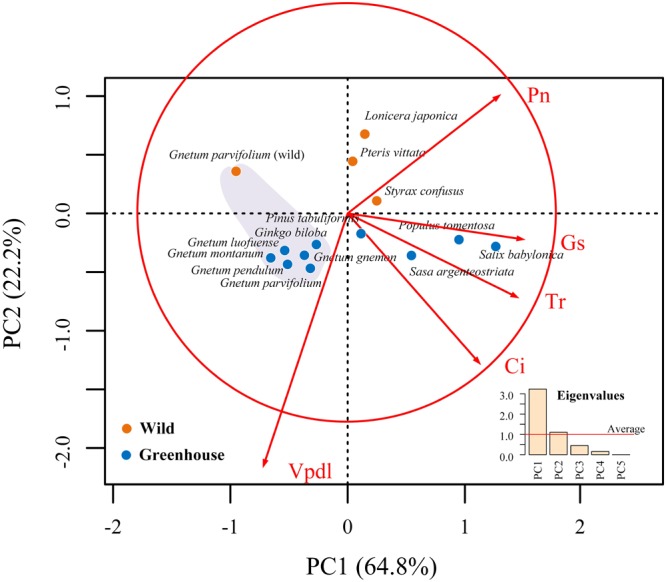
Principal component analyses (PCA) of five photosynthetic characters, i.e., net photosynthetic rate (Pn), transpiration rate (Tr), intercellular CO_2_ concentration (Ci), stomatal conductance (Gs), and leaf water deficit (Vpdl) among five *Gnetum* species and other seed plant representatives. Photosynthetic characters detected in the wild (orange) and in the greenhouse (blue). In PCA ordination, red circle represents the best-fit equilibrium contribution. Five red lines indicate that contribution of certain photosynthetic character is greater than average value. At the corner on right, different eigenvalues from PCA characters (PC1–PC5) are shown under Kaiser-Guttman criterion.

The results of PCA analyses (Figure 4) revealed that 87.0% of the variance in the data could be explained by the first two principal components, i.e., PC1 and PC2 (explaining 64.8% and 22.2%, respectively). The photosynthetic characters (Pn, Tr, Ci, Gs, and Vpdl) of the five *Gnetum* species clustered separately from those of other seed plant representatives. In addition, the photosynthetic characters of *G. parvifolium* measured in the wild were strongly differentiated from those of *Gnetum* species cultivated in the greenhouse (Figure 4).

**FIGURE 5:**
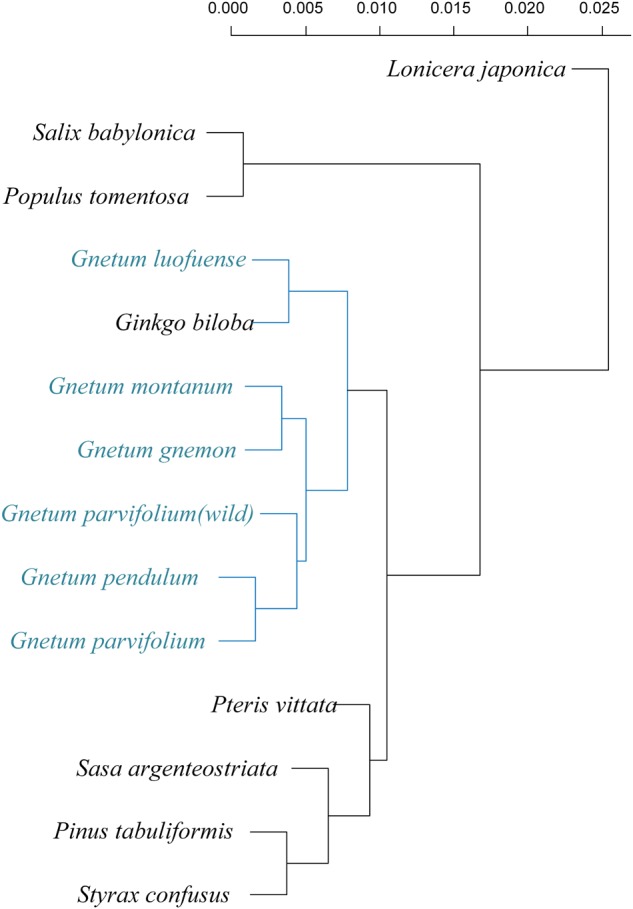
Phenetic relationships of five photosynthetic characters (arithmetic averages) as detected using unweighted pair-group method (UPGMA, coefficient = 0.89) among five species of *Gnetum* and eight seed plant representatives.

We compared the results of six clustering methods and found that the UPGMA method achieved the highest score of cophenetic correlation (0.89) (Figure 5; Supplementary Figure S1). Regardless of the clustering method used, the results suggested that photosynthetic characters of the five *Gnetum* species were similar to those of *Ginkgo* but considerably different from those of angiosperms and conifer representatives.

### Comparison of Chloroplast Genomes and Phylogenetic Reconstruction

**FIGURE 6:**
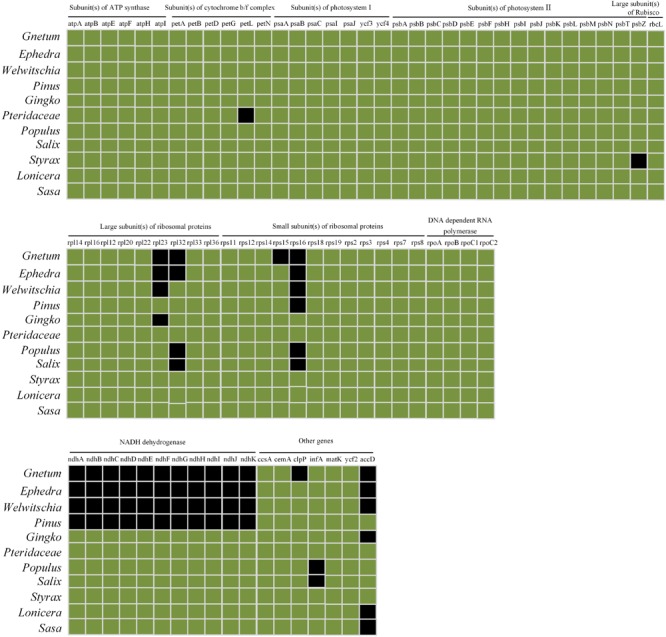
Comparison of coding genes among chloroplast genomes of Gnetales (i.e., *Gnetum, Ephedra*, and *Welwitschia*) and eight seed plant representatives including Dipsacales, Ericales, Ginkgoales, Malpighiales, Pinales, Poales, and Pteridales. Genes shown in black are absent from chloroplast genomes.

Comparisons among cpDNAs of *Gnetum* and other seed plant representatives (Figure 6) revealed that 17 coding genes were absent from the cpDNAs of *Gnetum* (Braukmann et al., 2009). Among the absent genes were *clpP* (encoding the ATP-dependent Clp protease proteolytic subunit), all 11 genes encoding NADH dehydrogenase, *accD, rpl23* (encoding ribosomal protein L23), *rpl32* (encoding ribosomal protein L32), *rps15*, and *rps16*. We found also that 15 and 14 genes were absent from the cpDNAs of *Ephedra* and *Welwitschia*, respectively. Specimens representing angiosperms formed a clade, which was sister to the clade representing gymnosperms, within which *Ginkgo biloba* was sister to the remaining group of species in this clade. The Gnetales were placed as the sister clade to the Pinaceae. However, the pattern of the cluster analyses based on photosynthetic characters seemed incongruent with the topology of the present phylogeny of the selected seed plants (Supplementary Figure S2). One possible explanation may be that insufficient plant species were included in the analyses. However, we note that the aim of constructing this tree was not to explain the phylogeny in this study, as mentioned in the method.

## Discussion

### Low Values for Photosynthetic Characters Within *Gnetum*

We conducted these experiments in the wild and in the greenhouse where the three most important factors for photosynthesis, i.e., light intensity, CO_2_ concentration, and temperature, were almost similar. The values of the photosynthetic characters Pn, Gs, and Tr were slightly lower for *G. parvifolium* in the wild than for *G. parvifolium* in the greenhouse (Table 3, Figure 1). Nevertheless, the photosynthetic features of *G. parvifolium* in the wild and in the greenhouse did not exhibit significant differences under the similar light conditions (for example, under high light intensity). We found consistent differences in photosynthetic characters between the arborescent species *G. gnemon* and Chinese lianoid species. In general, the capacities to utilize different light intensities were higher in lianoid species. Our results are also consistent with those of previous studies that detected low Pn, Gs, and Tr in *Gnetum* (Feild and Balun, 2008; Celis and Avalos, 2013). For example, the Pn was found to be 2.1–2.4 μmol m^-2^ s^-1^ CO_2_ in seedlings of *G. leyboldii* (Celis and Avalos, 2013), which is at the higher end of the range detected in the five *Gnetum* species we studied (Figure 1).

In the PCA analysis, we found that estimates of Gs made the largest contribution to PC1, followed by estimates of Tr and Pn, reflecting the remarkable differences in photosynthetic parameters between *Gnetum* and other land plants. Low values of Gs and Tr might reflect the restricted functions of syndetocheilie-type stomata in *Gnetum* leaves (Takeda, 1913). The Vpdl accounted for the majority of differentiation along PC2 and distinguished the photosynthetic characters of *G. parvifolium* measured in the field from those of other *Gnetum* species measured in the greenhouse. These differences might be due to differences in soil water availability and evaporation-transpiration rates under the two conditions. In addition, atmosphere and biota have likely co-evolved throughout history (Beerling et al., 2001; Igamberdiev and Lea, 2006; Franks and Beerling, 2009). High CO_2_ and sub-ambient O_2_ in the atmosphere can affect patterns of plant distribution and evolution (Fukao and Baileyserres, 2004; Wang D. et al., 2012; Haworth et al., 2013). Accordingly, we suggest that the photosynthetic capacities of extant *Gnetum* are probably inherited from their ancestors that evolved under the high global temperatures and CO_2_ density in the late Cretaceous to early Cenozoic (Zachos et al., 2001). These characteristics may thus provide an advantage to some species of this genus under scenarios of further global warming.

### Comparisons of Photosynthetic Characters and Chloroplast Genomes Between *Gnetum* and Other Seed Plants

The results of multivariate analyses revealed that some photosynthetic characters of *Gnetum* (Pn, Gs, Tr) differ markedly from those of other seed plants. Among the gymnosperms, *G. biloba* showed significantly different Pn, Tr, and Gs compared with those of *Gnetum* seedlings (Figure 3). This result was consistent with experimental data showing that 2-year-old *G. biloba* seedlings had a higher photosynthetic capacity than that of *Gnetum* (Zhang et al., 2005). The estimated values of photosynthetic characters were significantly larger in *P. tabuliformis* than in *Gnetum*, consistent with studies on other *Pinus* species (Di, 2009; Liu et al., 2009; Wang Z.X. et al., 2012). This is quite surprising, because conifers are characterized by lancelet or needle-shaped leaves that have a low Pn. However, their Pn was still higher than that of *Gnetum* leaves, which have eudicot morphology and pinnate leaf venation. The values of Pn, Tr, and Gs were consistently lower in *G. biloba* and *P. tabuliformis* than in the selected angiosperms (Figure 3), indicating that a low rate of photosynthesis might be a common characteristic of gnetophytes, conifers, and other gymnosperms. This topic should be investigated further in future studies.

The relationships among photosynthetic characters of other gymnosperms, comprising main lineages of conifers, cycads, *Ephedra* and *Welwitschia*, remain poorly understood. Morphologically, *Ephedra* has extremely reduced, small, linear leaves, and *Welwitschia* has giant strap-like leaves with numerous parallel longitudinal veins (Yang et al., 2015). Nevertheless, on the basis of the genomic data and morphological characters, we predict that low photosynthetic capacities could also characterize the two gnetalean genera whose habitats are in arid and semiarid areas of the world. Therefore, the ancestral status of photosynthetic characters in the Gnetales remains an open question.

We found that the values of photosynthetic characters, e.g., Pn, Tr, and Gs were significantly lower in *Gnetum* than in co-occurring lianoid angiosperms both in the wild and in the greenhouse. This can probably be ascribed to the different gross morphology and anatomical structures of *Gnetum* leaves. Compared with angiosperms, *Gnetum* and ferns have significantly lower densities of leaf veins (Zwieniecki and Boyce, 2014b). In addition, the veins in *Gnetum* leaves are arranged differently from veins in lianoid angiosperms, probably resulting in low efficiency of hydraulic transportation (Zwieniecki and Boyce, 2014a).

To adapt to low-light conditions beneath canopies in tropical forests, seedlings of lianoid angiosperms usually have a low light compensation and light saturation point (Yuan et al., 2016). After reaching maturity and being exposed to high-intensity light at the highest levels of canopies, seedlings of lianoid angiosperms can shift to high photosynthetic capacities and effective hydraulic conductance (Carter and Teramura, 1988). However, this is not the case in *Gnetum*. The results of the present study showed no significant differences in photosynthetic characters between *Gnetum* adults and seedlings. Therefore, the photosynthetic characters of *Gnetum* are ecophysiologically different from those of lianoid angiosperms. Nevertheless, the results of CO_2_ response experiment (Figure 2) revealed that *Gnetum* might have some potential for higher efficiency of photosynthesis. The maximum Pn value along the CO_2_ response curve was higher in *Gnetum* than in the co-occurring lianoid angiosperms. This result indicated that certain photosynthetic features of *Gnetum* have undergone evolution in parallel with those of angiosperms as an adaptation to tropical and subtropical forest environments where high humidity, high density of trunks or stems, and strong competition among plants are the prevailing conditions. Although *Gnetum* shares tropical biomes and habitats with lianoid angiosperms, *Gnetum* species do not show highly opportunistic and light-demanding ecophysiology. An intriguing question to address is the association between the ontogenetic flexibility of photosynthetic efficiency in angiosperms and the evolutionary success of this incredibly large clade of seed plants.

A previous study of Wu et al. (2009) shows that gnetophyte, including *G. parvifolium* in the genus of *Gnetum*, has specific loss of 18 genes common to cpDNAs of other land plants. Meanwhile, all 11 *ndh* genes encoding NADPH dehydrogenases are reported to be absent in the measured four *Gnetum* species (*G. gnemon, G. leyboldii* Tul., *G. ula* Brongn. and *Gnetum sp.*) (Braukmann et al., 2009). In the present study, we focused on investigating the relationship between low photosynthetic characters and gene loss of cpDNAs in *Gnetum*, based on the previous findings (Braukmann et al., 2009; Wu et al., 2009; Hou et al., 2016). The result showed that 17 coding genes are absent from cpDNAs of five *Gnetum* species, which has used in the work of Hou et al. (2016), compared with those selected seed plant representatives (Figure 5 and 6; Supplementary Figure S2). Among the lost genes are those encoding subunits of ATP synthase, cytochrome b/f complex, photosystem I and II, and the large subunit of Rubisco, all of which are involved in particular pathways of photosynthesis (Salah et al., 2016). In addition, we found that all genes encoding NADPH dehydrogenases are absent from the cpDNAs of *Gnetum*. These genes are also absent from cpDNAs of *Pinus* (Braukmann et al., 2009; Wu et al., 2009), suggesting support for the Gnepine hypothesis that places the Gnetales as the sister lineage of Pinaceae. NADPH dehydrogenases are an important part of the membrane protein complex that mediates the uptake of CO_2_, transport of photosystem I-dependent cyclic electrons, and cellular respiration (Ueda et al., 2012; Yamori et al., 2015). As regards to these photosynthetic functions, therefore, the absence of genes encoding NADPH probably affects the efficiency of energy conversion similarly in both *Gnetum* and *Pinus* (Wu et al., 2009). However, we should not overlook the closer relationship between *Gnetum* and *Ginkgo* than between *Gnetum* and *Pinus*, as suggested by PCA and cluster analyses of the whole set of photosynthetic characters used in ourstudy (Figure 4 and 5). These results indicate the likely low impact of the lack of these NADPH genes and their functions on the overall photosynthetic characters of plants, at least at the deep evolutionary scale of the three main gymnosperm lineages.

## Conclusion

The results of this study further corroborate that *Gnetum* species have low Pn, Gs, and Tr. In addition, we found that multiple chloroplast genes that are believed to be essential for photosynthesis are absent from *Gnetum*, probably resulting in its particular evolutionary history. The other gnetophytes, *Ephedra* and *Welwitschia*, which have been adapted to arid and semiarid living habitats, lack the same chloroplast genes. This finding suggests that low photosynthetic capacity may also be a characteristic of these two genera, and the unique evolutionary history could be a feature of the entire group. Nevertheless, further research is required to investigate the molecular mechanisms underlying the low photosynthetic capacity in gnetophytes. It will also be interesting to compare gross morphology and anatomical structures of pinnate-veined leaves and vessels in association with transcriptome differences between *Gnetum* and angiosperms. Accordingly, these analyses of photosynthetic, physiological, and morphological characters of *Gnetum* in combination with the particular structure of its cpDNA provide a new perspective on the evolutionary history of the Gnetales.

## Author Contributions

ND and CH make the major contribution to this article, and other authors also make considerable contributions to this article.

## Conflict of Interest Statement

The authors declare that the research was conducted in the absence of any commercial or financial relationships that could be construed as a potential conflict of interest.

## Footnotes

1 https://www.r-project.org/

2 www.ncbi.org

## Supplementary Material

FIGURE S1 Cluster analyses of five photosynthetic characters i.e., net photosynthetic rate (Pn), transpiration rate (Tr), intercellular CO_2_ concentration (Ci), stomatal conductance (Gs), and leaf water deficit (Vpdl) among five *Gnetum* species and eight representatives of other land plants using six different methods. Numbers in red are values of cophenetic correlation coefficient.

FIGURE S2 Phylogenetic tree inferred from literature showing evolutionary relationships across seed plants and within *Gnetum* based on chloroplast genome data (Won and Renner, 2006; Wu et al., 2007; Hou et al., 2016), and presence/absence of particular genes in chloroplast genomes of seed plants. Genes shown in black in right panel are absent. For complete list of genes in panel, see Supplementary Table S1. Complete chloroplast genome sequence data for correspondent taxa were obtained from GenBank.

TABLEE S1 Complete list of protein-coding genes in chloroplast genomes included in this study. See Table 2 for GenBank accession numbers.
