# Supplementary figures and images for: Significance of Photosynthetic Characters in the Evolution of Asian Gnetum (Gnetales)
Source: Front Plant Sci. 2019 Feb 5;10:39. doi: 10.3389/fpls.2019.00039 (PMC6370715; doi:10.3389/fpls.2019.00039)

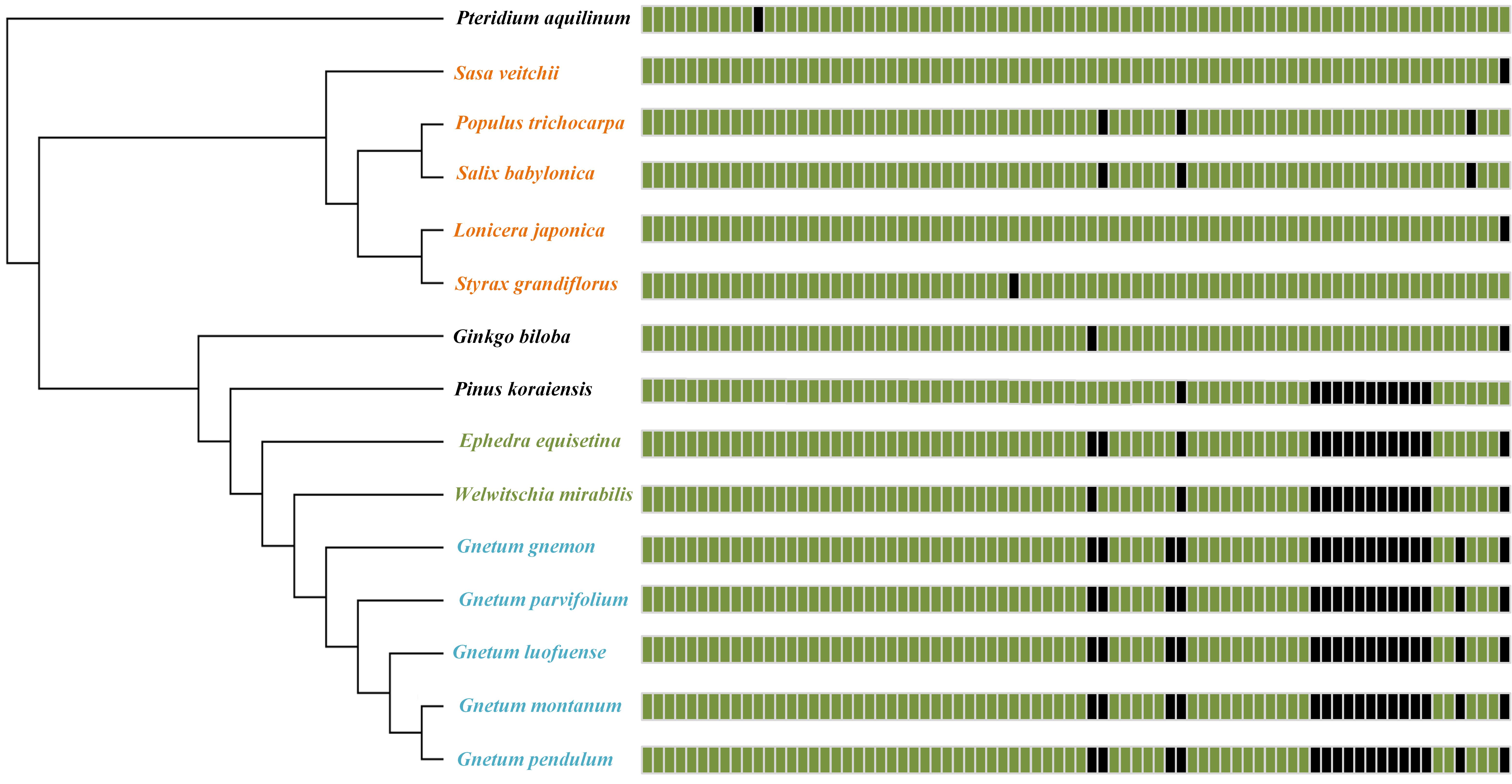

Supplement: FIGURE S2 — Phylogenetic tree inferred from literature showing evolutionary relationships across seed plants and within Gnetum based on chloroplast genome data (Won and Renner, 2006; Wu et al., 2007; Hou et al., 2016), and presence/absence of particular genes in chloroplast genomes of seed plants. Genes shown in black in right panel are absent. For complete list of genes in panel, see Supplementary Table S1. Complete chloroplast genome sequence data for correspondent taxa were obtained from GenBank. [file Image_2.JPEG]
